# A PHABULOSA-Controlled Genetic Pathway Regulates Ground Tissue Patterning in the *Arabidopsis* Root

**DOI:** 10.1016/j.cub.2020.10.038

**Published:** 2021-01-25

**Authors:** Gaia Bertolotti, Simon Josef Unterholzner, Daria Scintu, Elena Salvi, Noemi Svolacchia, Riccardo Di Mambro, Veronica Ruta, Francisco Linhares Scaglia, Paola Vittorioso, Sabrina Sabatini, Paolo Costantino, Raffaele Dello Ioio

**Affiliations:** 1 Dipartimento di Biologia e Biotecnologie, Laboratory of Functional Genomics and Proteomics of Model Systems, Università di Roma, Sapienza - via dei Sardi, 70, 00185 Rome, Italy; 2 Faculty of Science and Technology, Free University of Bozen-Bolzano, Piazzale Università, 5, 39100 Bolzano, Italy; 3 Department of Biology, University of Pisa, via L. Ghini, 13, 56126 Pisa, Italy; 4 Center of Nuclear Energy in Agriculture (CENA), University of São Paulo-USP, São Paulo, Brazil

**Keywords:** homeodomain leucin zipper III, cyclin D6;1, CYCD6;1, middle cortex, gibberellin, ground tissue, root development, microRNA

## Abstract

In both animals and plants, development involves anatomical modifications. In the root of *Arabidopsis thaliana*, maturation of the ground tissue (GT)—a tissue comprising all cells between epidermal and vascular ones—is a paradigmatic example of these modifications, as it generates an additional tissue layer, the middle cortex (MC).^1^, ^2^, ^3^, ^4^ In early post-embryonic phases, the *Arabidopsis* root GT is composed of one layer of endodermis and one of cortex. A second cortex layer, the MC, is generated by asymmetric cell divisions in about 80% of *Arabidopsis* primary roots, in a time window spanning from 7 to 14 days post-germination (dpg). The cell cycle regulator CYCLIN D6;1 (CYCD6;1) plays a central role in this process, as its accumulation in the endodermis triggers the formation of MC.^5^ The phytohormone gibberellin (GA) is a key regulator of the timing of MC formation, as alterations in its signaling and homeostasis result in precocious endodermal asymmetric cell divisions.^3^^,^^6^^,^^7^ However, little is known on how GAs are regulated during GT maturation. Here, we show that the HOMEODOMAIN LEUCINE ZIPPER III (HD-ZIPIII) transcription factor PHABULOSA (PHB) is a master regulator of MC formation, controlling the accumulation of CYCD6;1 in the endodermis in a cell non-autonomous manner. We show that PHB activates the GA catabolic gene *GIBBERELLIN 2 OXIDASE 2* (*GA2ox2*) in the vascular tissue, thus regulating the stability of the DELLA protein GIBBERELLIN INSENSITIVE (GAI)—a GA signaling repressor—in the root and, hence, *CYCD6;1* expression in the endodermis.

## Highlights

• PHB regulates cell non-autonomously the timing of MC formation
• A time-dependent rise of *PHB* expression controls the CYCD6;1 switch in the GT
• PHB regulates GAI stability modulating GA levels
• PHB regulates root GA levels activating *GA2ox2* expression in the vasculature

## Graphical Abstract

**Figure:**
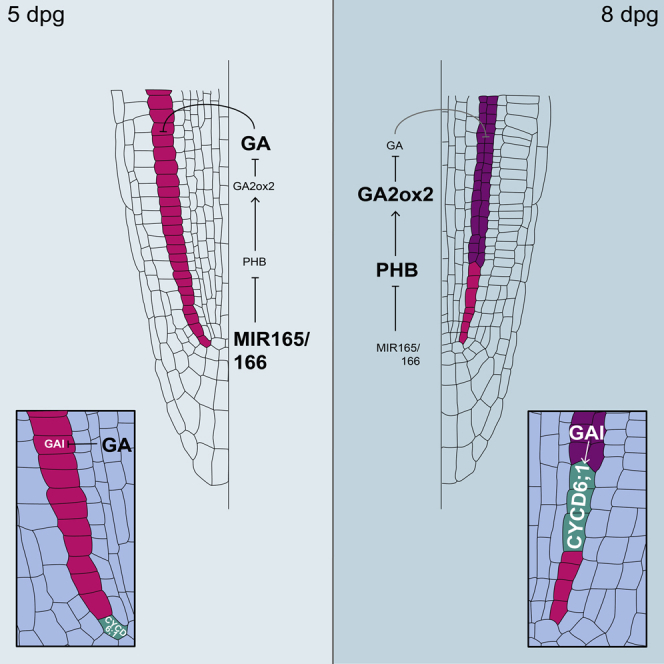

## Teaser

Bertolotti, Unterholzner et al. show that a time-dependent threshold of the transcription factor PHB governs the timing of middle cortex formation in the *Arabidopsis* root. PHB promotes *CYCD6;1* expression in the endodermis cell non-autonomously by reducing gibberellin (GA) levels in the vascular tissue and, hence, stabilizing the GAs repressor GAI.

## Results and Discussion

### PHB and PHV Control MC Formation via the Regulation of the *CYCLIN D6;1* (*CYCD6;1*) Expression Domain

**Figure 1:**
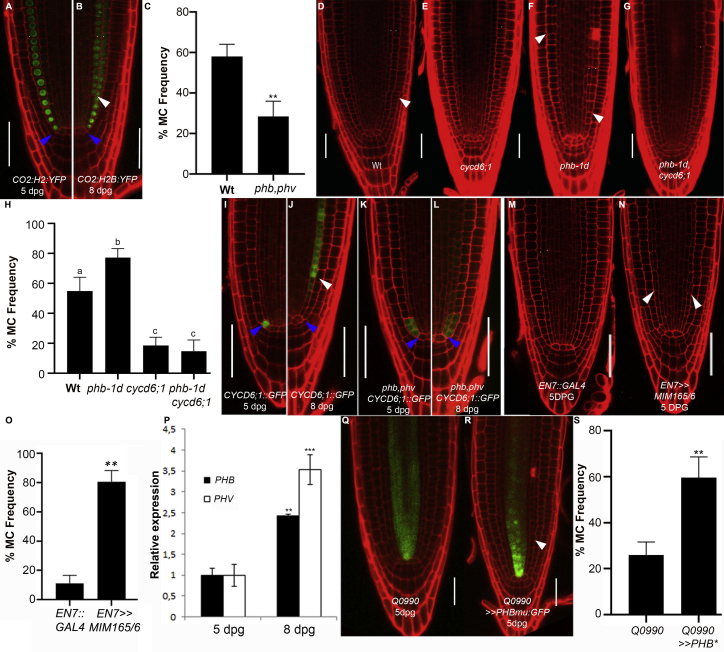
PHB Regulates MC Formation Cell Non-autonomously (A and B) Confocal images of *CO2::H2B:YFP* at 5 (A) and 8 (B) dpg. (C) Histogram depicting the percentage of plants showing MC formation in WT, *phb-13*, and *phv-11* mutants at 8 dpg. (D–G) Confocal images of 8 dpg old root meristems of WT (D), *cycd6;1-1* (E), *phb-1d* (F), and *phb-1d, cycd6;1-1* (G). (H) Histogram reporting the percentage of MC formation in WT, *phb-1d*, *cycd6;1-1*, and *phb-1d, cycd6;1-1*. p < 0.005; ANOVA. (I–L) Confocal images of *CYCD6;1::GFP:GUS* and of *phb-13, phv-11, CYCD6;1::GFP:GUS* at 5 (I and K) and 8 dpg (J and L). (M and N) Confocal images of root meristems of *EN7::GAL4* (M) and *EN7>>MIM165/6* (N) at 5 dpg. (O) Histogram depicting the percentage of MC formation in *EN7::GAL4* and *EN7>>MIM165/6* at 5 dpg. (P) Relative expression of *PHB* and *PHV* in WT plants at 5 and 8 dpg. N = 3. (Q and R) Confocal image of *Q0990* and *Q0990>>PHBmu:GFP* root meristems at 5 dpg. (S) Histogram depicting the percentage of MC formation in *Q0990* and *Q0990>>PHBmu:GFP* at 5 dpg. Scale bars, 50 μm; white arrowheads, MC; blue arrowheads, CEI. Student’s t test (^∗^p < 0.05; ^∗∗^p < 0.01; ^∗∗∗^p < 0.005 ). n = 20, N = 3. Different letters show statistical significance. Error bars: SD. See also Figure S1.

In *Arabidopsis*, expression of *PHABULOSA* (*PHB*) and of its redundant homologous *PHAVOLUTA* (*PHV*) is restricted to the vascular tissue due to the repressive activity of microRNA165 (miRNA165) and 166 in the ground tissue (GT).^8^, ^9^, ^10^ We have recently shown that miR165- and 166-resistant mutants of PHB and PHV (*phb-1d* and *phv-1d*, respectively), where *PHB* and *PHV* are present also in the GT, have supernumerary cortex formation already during early phase of root development, suggesting that these transcription factors regulate GT patterning.^11^ Because *Arabidopsis* plants acquire an additional cortical layer in late post-embryonic root development (Figures 1A and 1B), we assessed whether PHB and PHV control middle cortex (MC) formation analyzing MC development in *phb,phv* loss-of-function plants (*phb-13, phv-11*).^12^ Under our conditions, at 8 days post-germination (dpg), about 55% of wild-type (WT) plants start to develop MC, whereas only 25% of the *phb,phv* roots show a second cortical layer (Figure 1C),^13^ suggesting that PHB and PHV may control MC development.

The *CYCD6;1* gene is necessary for MC formation.^5^ We have recently shown that *phb-1d* roots have higher expression of *CYCD6;1* in the GT.^11^ To assess whether PHB controls the number of cortical layers via CYCD6;1, we generated a *phb-1d,cycd6;1-1* double mutant and analyzed GT development at 8 dpg. Only about 15% of *phb-1d,cycd6;1-1* and *cycd6;1-1* roots showed an additional cortical layer as compared to 75% of *phb-1d* (Figures 1D–1H), thus suggesting that PHB requires CYCD6;1 activity to promote MC formation.

*CYCD6;1* shows a maximum of expression in the cortex/endodermis initial (CEI) and in its daughter cell (CEID) from embryogenesis up to 5 dpg, although subsequently, it is predominantly expressed in the endodermis. To assess whether PHB and PHV control this time-dependent variation in *CYCD6;1* expression, we analyzed *CYCD6;1* in WT and *phb, phv* plants harboring the *CYCD6;1* promoter fused to the *GREEN FLUORESCENT* and *GLUCURONIDASE* genes (*CYCD6;1::GFP:GUS*). At 5 dpg, in WT roots, GFP signal is detectable in the CEI, CEID, endodermis, and cortex, although at 8 dpg, it is mostly present in the endodermis and in newly formed MC (Figures 1I and 1J). At the contrary, in *phb,phv* roots, the GFP signal is detectable in the CEI, CEID, and endodermis both at 5 dpg and at 8 dpg (Figures 1K and 1L).

Altogether, these data suggest that PHB and PHV regulate MC formation controlling the timing of *CYCD6;1* expression. As PHB and PHV are both sufficient to promote cortex formation,^11^ we focused our studies on PHB.

miR165a, 166a, and 166b act from the endodermis to control *PHB* and *PHV* expression in the GT and in the vasculature.^2^^,^^11^ qRT-PCR on WT roots and GFP fluorescent signal of the transcriptional reporters of MIR165A and 166a (*MIR165A::GFP* and *MIR166A::GFP*) revealed that *pre-miR165a* and *pre-mir166a* decrease between 5 and 8 dpg (SD1). To understand whether this decrease results in precocious MC formation, we knocked down miR165 and 166 in the endodermis, expressing *MIMICRY165/6* (*MIM165/6*)^14^ under the control of the *ENDODERMIS7* (*EN7*) promoter, driving expression specifically in CEI, CEID, and endodermis.^11^^,^^13^ *EN7>>MIM165/6* plants show early MC formation (Figures 1M–1O; SD1), suggesting that decreased levels of miR165/6 govern MC timing formation. PHB/PHV/miR165/166 module controls vasculature patterning other than MC formation, nonetheless neither *phb,phv*^8^ nor *EN7>>MIM165/6* (SD1) show vascular defects, suggesting that the two events might be independent.

As miR165 and 166 levels decrease at 8 dpg, we thought that *PHB* expression might expand in the GT. Analysis of plants carrying a translational GFP reporter fusion (*PHB-GFP*) revealed that no *PHB* expression could be detected in the GT at 8 dpg (SD1), indicating that PHB might act cell non-autonomously from the vascular tissue to promote MC formation.

*PHB* is expressed in the vasculature already during embryogenesis but promotes MC formation only at 7 to 8 dpg.^3^ Because *phb-1d* mutants have higher levels of *PHB* mRNA than the WT and show precocious MC formation,^15^ we hypothesized that, in WT plants, *PHB* might increase between 5 and 8 dpg: indeed, qRT-PCR indicates that *PHB* level increases between 5 and 8 dpg (Figure 1P). To understand whether increased *PHB* expression in the vasculature is responsible for MC formation, we overexpressed a miRNA-insensitive version of PHB fused to the GFP (*PHBmu:GFP*) specifically in this domain from early stages of root development utilizing the GAL4/upstream activating sequence (UAS) transactivation system (*Q0990,UAS::PHBmu:GFP*).^9^ Interestingly, *Q0990>>PHBmu:GFP* roots show MC formation already at 5 dpg (Figures 1Q–1S), supporting the notion that increased *PHB* levels in the vasculature promote MC formation cell non-autonomously.

Because *CYCD6;1* expression in the endodermis is a necessary requirement for MC formation, we hypothesized that *PHB* might promote *CYCD6;1* expression in the endodermis cell non-autonomously from the vasculature. Thus, we generated *Q0990>>PHBmu:GFP*, *CYCD6;1::GFP:GUS* plants. *Q0990>>PHBmu:GFP* plants show *CYCD6;1* expression in the endodermis already at 5 dpg, suggesting that an increase of *PHB* in the vasculature is sufficient to control the switch of *CYCD6;1* expression from the CEI/D to the endodermis (SD1).

These results suggest that increased *PHB* expression in the vasculature is sufficient to control the timing of MC formation regulating the switch of *CYCD6;1* expression from the CEI/D to the endodermis.

### PHB Regulates GAI Stability

**Figure 2:**
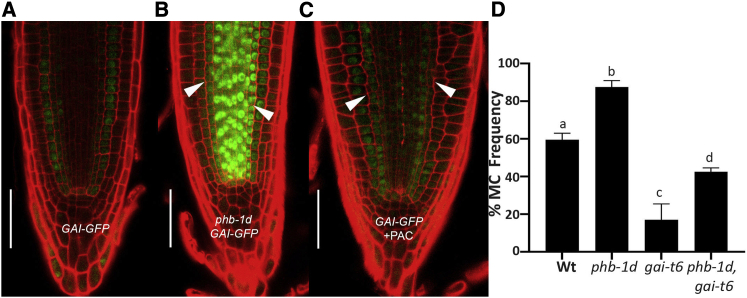
PHB Promotes GAI Stabilization (A–C) Root meristems of *GAI-GFP* (A); *phb-1d, GAI-GFP* (B); and PAC-treated (50 μM; 24 h) *GAI-GFP* (C) at 5 dpg. Scale bars, 50 μm; white arrowheads, MC. (D) Histogram reporting the percentage of MC formation in WT; *phb-1d*, *gai-t6*; and *phb-1d, gai-t6.* Student’s t test. Different letters show statistical significance. n = 20, N = 3. Error bars: SD; p < 0.005; ANOVA. See also Figure S2.

Gibberellins (GAs) are key regulators of MC timing formation; high level of GA activity, achieved through the degradation of the DELLA proteins GIBBERELLIC ACID INSENSITIVE (GAI) and REPRESSOR OF GAI (RGA), represses MC formation.^3^^,^^16^ To assess whether PHB regulates *GAI* and *RGA*, we analyzed the translational fusions *GAI-GFP* and *RGA-GFP* in *phb-1d* roots. This revealed that *GAI* is expressed at higher level and with an expanded domain in *phb-1d* compared to WT roots: in *phb-1d* roots, the signal is enhanced and present in both the GT and the vasculature, although in WT roots, *GAI-GFP* fluorescence was detectable only in the GT (Figures 2A and 2B). In contrast, *RGA* expression pattern in *phb-1d* remains unchanged (SD2).

GA activity is fine-tuned by a negative-feedback loop with DELLA proteins, such as GAI: high GA levels promote GAI degradation via the proteasome pathway, enabling the expression of GA-dependent genes; conversely, GAI represses the response to GA, inhibiting the activity of GA-dependent transcription factors.^17^, ^18^, ^19^ To understand whether PHB controls GAI transcription, we measured *GAI* mRNA level in *phb-1d* via qRT-PCR; *GAI* mRNA level does not vary in this background (SD2), suggesting that PHB controls GAI abundance at the protein level. Consistently with this, *GAI-GFP* plants treated with the GA biosynthesis inhibitor paclobutrazol (PAC) showed the GFP signal is present in both the root GT and the vasculature (Figures 2A–2C), similarly to *phb-1d* roots. To establish whether PHB regulates MC formation through the control of GA levels, we treated *phb,phv* mutants for 48 h with PAC. We observed that PAC treatment was sufficient to promote MC formation in *phb,phv* roots at 5 dpg (SD2), suggesting that the decreased MC formation in *phb,phv* is due to high GA levels. These results indicate that PHB promotes GAI protein stability via the control of GA levels.

To assess whether GAI is necessary to regulate MC development, we analyzed MC formation in the loss-of-function mutants *gai-t6*, *gai-2*, and *gai-3*: at 8 dpg, only about 20% of the roots from the three *gai* mutants show formation of MC (Figure 2C; SD3), suggesting that GAI is required for the correct development of the MC.

The control of GA homeostasis is required to regulate the timing of *CYCD6;1* expression in the endodermis.^16^^,^^20^ Indeed, PAC treatment on *CYCD6;1::GFP:GUS* plants is sufficient to promote early expression of this gene in the endodermis at 5 dpg, causing a precocious MC formation (SD3).^3^^,^^21^ Similarly to PAC-treated plants, we observed that the *gai-1* gain-of-function mutant—where GAI is insensitive to the GA-dependent degradation^22^—forms MC earlier and accumulates *CYCD6;1::GFP:GUS* signal in the endodermis already at 5 dpg (SD3), suggesting that the increase of GAI stability is sufficient to promote *CYCD6;1* in the endodermis and, in turn, MC formation.

We then tested whether GAI activity mediates PHB-dependent regulation of MC formation. To this end, we generated *gai-t6,phb-1d* plants; loss of GAI partially rescues the phenotype of *phb-1d* mutants (Figure 2D), suggesting that PHB requires GAI activity to promote MC formation.

### PHB Regulates GA Homeostasis via GA2ox2

GAI stability depends on GA levels, which in turn depend on the rate of GA catabolism and synthesis.^23^ GA synthesis is controlled by the GA3ox and Ga20ox enzymes, but neither of these genes is expressed in the root meristem.^24^^,^^25^ GA degradation depends on the activity of the GIBBERELLIN 2 OXIDASE (GA2ox) dioxygenases,^26^^,^^27^ among which *GA2ox2* is expressed, as *PHB*, mostly in the vasculature (SD4).^28^ Thus, we hypothesized that PHB might promote GAI stability via the control of *GA2ox2* expression.

**Figure 3:**
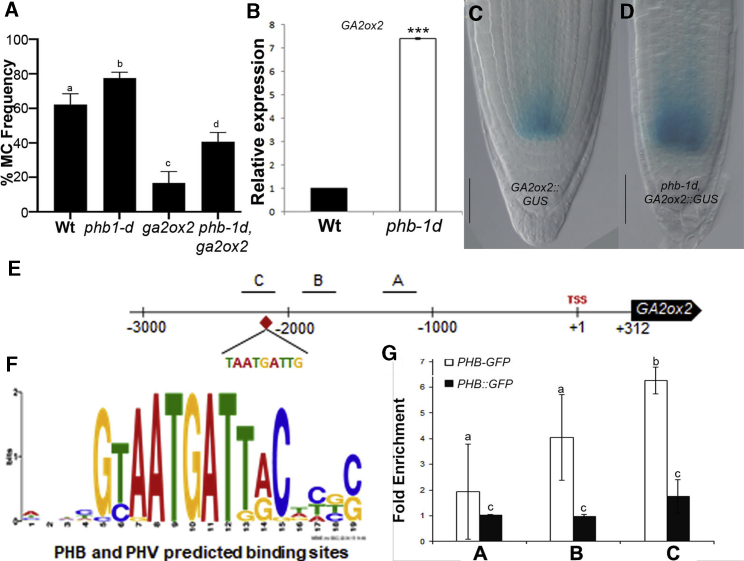
PHB Directly Regulates *GA2ox2* Expression (A) Histogram depicting the percentage of MC formation in WT; *phb-1d, ga2ox2-1*; and *phb-1d, ga2ox2-1* plants at 8 dpg. p < 0.005; ANOVA. Different letters show statistical significance. (B) *GA2ox2* relative expression in WT and *phb-1d* plants. (C and D) Root meristems of *GA2ox2::GUS* (C) and *phb-1d, GA2ox2::GUS* (D) plants at 5 dpg. Scale bars, 50 μm. (E and F) *GA2ox2* promoter illustration. TSS indicates the transcriptional start site (+1) and the fragments used as probes for the ChIP experiment are marked with A (−1,186/−1,214 bp), B (−1,755/−1,909 bp), and C (−2,123/−2,271 bp). The red rhombus indicates the putative binding site of PHB and PHV, TAATGATTG (PlantPAN2.0) illustrated in (F). (G) ChIP experiment using root meristems of *PHB-GFP* at 8 dpg. Fold enrichment of *PHB-GFP* on the indicated fragments A, B, and C was determined by qRT-PCR and calculated as ratio of anti-GFP IP to control beads immunoprecipitation (IP) of each independent replicate. *UBQ10* was used for normalization. (A and B) n = 20, N = 3; Student’s t test (^∗^p < 0.05; ^∗∗^p < 0.01; ^∗∗∗^p < 0.005). Error bars: SD. See also Figure S3.

We first found that GA2ox2 is required for MC development, as only 20% of *ga2ox2-1* loss-of-function mutants^29^ show MC formation at 8 dpg (Figure 3A). Interestingly, in *ga2ox2-1*, a strong reduction of GA levels, due to 48-h PAC treatment, results in MC formation at 5 dpg (SD2). This suggests that *ga2ox2-1* root phenotype is due to increased GA levels.

To assess whether PHB promotes *GA2ox2* expression, we analyzed *GA2ox2* mRNA level in *phb-1d* via qRT-PCR and found higher levels of *GA2ox2* compared to the WT (Figure 3B). Moreover, analysis of the transcriptional reporter *GA2ox2::GUS* showed that the *GA2ox2* expression domain is wider in *phb-1d* than in the WT (Figures 3C and 3D).

To evaluate whether *GA2ox2* is a PHB direct target, we first performed an *in silico* analysis^30^ that revealed a canonical HOMEODOMAIN LEUCINE ZIPPER III (HD-ZIPIII) recognition site in the *GA2ox2* promoter (Figures 3E and 3F). Therefore, we performed a chromatin immunoprecipitation (ChIP) assay from 8-dpg-old *PHB-GFP* and *PHB::GFP* roots: ChIP-qPCR revealed that the fragment including the putative HD-ZIPIII site was enriched in the GFP-IP chromatin of *PHB-GFP*, but not in *PHB::GFP*, indicating that PHB-GFP binds directly to the *GA2ox2* promoter (Figure 3G).

We then investigated whether PHB requires GA2ox2 activity to control MC formation, analyzing GT development in *phb-1d,ga2ox2-1* double mutants. At 8 dpg, 75% of *phb-1d* roots, as opposed to 35% of *phb-1d,ga2ox2-1* plants, show an additional cortical layer (Figure 3A), indicating that PHB requires GA2ox2 to promote MC formation.

**Figure 4:**
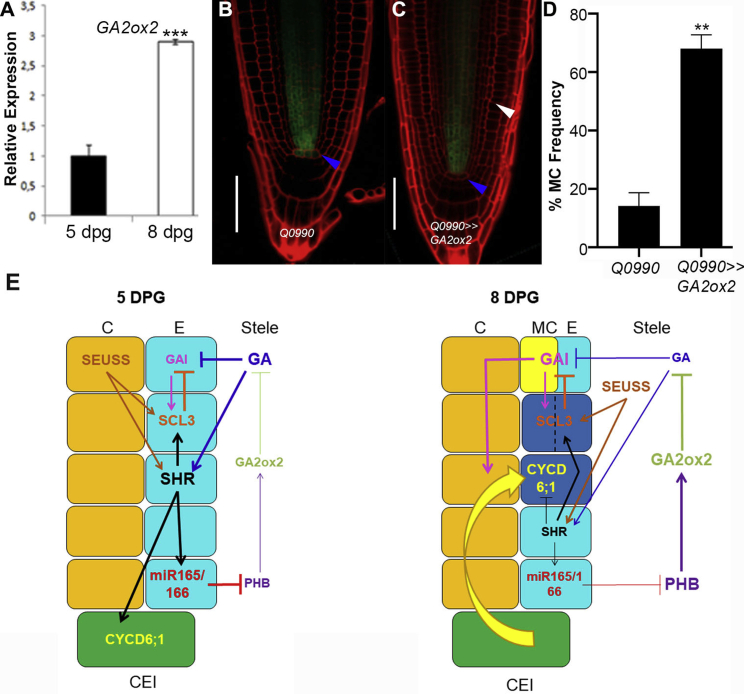
GA2ox2 Regulates MC Formation Cell Non-autonomously (A) Relative expression of *GA2ox2* in 5 and 8 dpg old WT plants. N = 3. (B and C) Confocal images of meristems of *Q0990* (B) and *Q0990>>GA2ox2* plants at 5 dpg (C). Scale bars, 50 μm; white arrowhead indicates MC formative asymmetric division; blue arrowheads indicate the CEI. (D) Histogram depicting the percentage of MC formation in *Q0990* and *Q0990>>Ga2ox2* at 5 dpg. n = 20, N = 3. (A and D) n = 3; Student’s t test (^∗∗^p < 0.01; ^∗∗∗^p < 0.005). Error bars: SD. (E) Model: PHB levels increase between 5 and 8 dpg, resulting in increased *GA2ox2* expression. Increased GA2ox2 levels promote the degradation of GAs in the vasculature, stabilizing GAI protein. GAI directs the accumulation of CYCD6;1 in the endodermis, promoting MC formation. Decrease levels of GAs after 5 dpg dampens SHR levels that regulate miR165 and 166 and SCL3 that in turn attenuate PHB expression and GAI activity, respectively. Orange, cortex (C); cyan, endodermis (E); yellow, middle cortex (MC); green, cortex/endodermis initial (CEI); blue, periclinally dividing cells (dashed line). Yellow arrow indicates the CYCD6;1 switch. See also Figure S4.

As *PHB* mRNA increases between 5 and 8 dpg, we wondered whether also *GA2ox2* mRNA might increase in this time frame in a PHB- and PHV-dependent fashion: qRT-PCR showed that indeed it does, although the expression pattern does not change (Figure 4A; SD4). This suggests that, similarly to PHB, the increase in *GA2ox2* expression in the vasculature may be sufficient to promote MC formation. To verify this possibility, we increased *GA2ox2* levels in the vasculature during early stages of root development, generating *Q0990*,*UAS::GA2ox2* plants (*Q0990>>GA2ox2*): roots of these plants show MC formation already at 5 dpg (Figures 4B–4D), confirming that an increased *GA2ox2* expression in the vasculature is sufficient to promote MC formation.

Our data indicate that increase of *PHB* expression regulates the timing of MC formation by controlling GA homeostasis in the vasculature; PHB promotes *GA2ox2* expression in this tissue, regulating GAs catabolism in the root and, hence, the timing of *CYCD6;1* expression in the endodermis cell non-autonomously, and the GA2ox2-dependent decrease of GA level stabilizes GAI, thus promoting *CYCD6;1* accumulation in the endodermis and, consequently, MC formation (Figure 4E). PHV might work similarly to PHB, as *PHV* mRNA increases between 5 and 8 dpg (Figure 1P).

Our data suggest that there might be a threshold of PHB/PHV levels resulting in a precise temporal regulation of GA levels to promote MC formation. SHORTROOT (SHR) and SCARECROW (SCR) promote *miR165* and *miR166* expression in the GT.^8^^,^^9^^,^^31^ GAs promote *miR165* and *miR166* expression and SHR accumulation in the endodermis, and GA activity decreases after 5 dpg.^3^^,^^16^^,^^32^^,^^33^ We propose that the decrease in miR165 and 166 levels after 5 dpg may depend on the reduction in GA activity, and hence SHR accumulation, causing an increase of *PHB/PHV* expression (Figure 4E). Consistently, GA treatments decrease *PHB* mRNA in WT roots (SD4).

GA activity in the root meristem depends on the coordinated action of SEUSS (SEU), SHR, SCR, and SCARECROWLIKE3 (SCL3).^3^^,^^16^^,^^34^^,^^35^ SEU induces *SHR*, *SCR*, and *SCL3*, and this latter is also a direct target of the SHR/SCR complex. SCL3 promotes GA signaling, dampening activity of DELLA proteins; GAs repress *SCL3* expression, generating a negative feedback loop that fine-tunes GA activity in roots (Figure 4E).^3^^,^^16^^,^^34^ The PHB-dependent GA homeostasis control might be coordinated with the SEU/SHR/SCR/SCL3 pathway. Consistently, the *scl3,phb,phv* triple mutant resembles *phb,phv* mutant at 8 dpg, suggesting that PHB and PHV are epistatic to *SCL3* (SD4). PHB and PHV might control SCL3 either via the GA-dependent regulation of SHR level in the endodermis or through GAI-dependent regulation of SCL3.^21^^,^^36^^,^^37^ Future studies will unravel how those two pathways integrate to mediate proper MC development.

The *phb-1d,ga2ox2-1* double mutant shows a partially restored root WT phenotype, whereas *phb-1d,cycd6;1-1* root resembles *cycd6;1-1* ones. This suggests that PHB might act not only via the cell non-autonomous regulation of GA levels to promote *CYCD6;1* expression in the endodermis^9^^,^^11^ but also cell-autonomously via some other yet unidentified mechanisms. The presence of two different mechanisms might be at the base of the interspecific variability in GT patterning; plants like *Arabidopsis*, where *PHB* expression is confined in the vasculature, acquire post-embryonically an additional cortical layer, whereas other species, such as *Cardamine hirsuta*, where *PHB* is expressed in the GT, show multiple cortical layers since embryogenesis.^11^^,^^38^ A PHB-dependent cell non-autonomous mechanism might be sufficient for species whose roots acquire MC only in late stages of development, whereas in roots of species having multiple cortical layers since embryogenesis, this mechanism could be combined with a cell-autonomous one.

## STAR★Methods

### Key Resources Table

**Table:** 

REAGENT or RESOURCE	SOURCE	IDENTIFIER
**Bacterial and Virus Strains**		

*Escherichia coli* DH5α	N/A	N/A
*Agrobacterium tumefaciens* GV3101	N/A	N/A

**Chemicals, Peptides, and Recombinant Proteins**		

Murashige & Skoog Medium	Duchefa	Cat# M0221
MES hydrate	Duchefa	Cat# M1503
Plant-agar	Duchefa	Cat# P1001
Sucrose	Duchefa	Cat# S0809
Kanamycin	Sigma-Aldrich	Cat# K1377
Rifampicin	Duchefa	Cat# R0146
Tetracycline	Duchefa	Cat# T0150
Gentamicin	Duchefa	Cat# G0124
Streptomycin	Duchefa	Cat# S0148
Spectinomycin	Duchefa	Cat# S0188
Phosphinothricin	Sigma-Aldrich	Cat# 77182-82-2
Phusion High-Fidelity DNA Polymerase	New England Biolabs	Cat# M0530S
trans-Zeatin	Sigma-Aldrich	Cat# Z0876
Dexamethasone	Sigma-Aldrich	Cat# D4902
Hpy188III	NEB	Cat# R0622S
Complete protease inhibitor cocktail	Roche	Cat# 11697498001
x-GlcA	Duchefa	Cat# X1405.1000
Dimethyl-sulfoxide	Sigma-Aldrich	Cat# 67-68-5
Ethanol	Sigma-Aldrich	Cat# 64-17-5
Na2HPO4	Duchefa	Cat# 10028-24-7
NaH2PO4	Carlo Erba	Cat# 7558-80-7
K3 Fe(CN)6	Sigma-Aldrich	Cat# 13746-66-2
K4Fe(CN)6	Sigma-Aldrich	Cat# 14459-95-1
Chloral Hydrate	Acros Organics	Cat# 302-17-0
Glycerol	Sigma-Aldrich	Cat# 56-81-5
Paclobutrazol	Duchefa	Cat# P0922.0500
GA4+7	Duchefa-Biochemie	Cat# G0938
Glycine	BIORAD	Cat# 1610718
Formaldehyde solution 37%	Sigma-Aldrich	Cat# 252549
Miracloth	Merck-Millipore	Cat# 475855
Hydrocloric acid 37%	Fisher scientific	Cat# 1298971
Magnesium chloride	Carlo Erba	Cat# 459337
DL-1,4-Dithiothreitol	Acros Organics	Cat# 327190100
Triton X-100	Acros Organics	Cat# 215680010
Ethylenediaminetetraacetic acid disodium salt	Carlo Erba	Cat# 405497
Sodium lauryl sulfate	Carlo Erba	Cat# P7600517
Sodium chloride	Duchefa-Biochemie	Cat# S0520.5000
GFP Trap_A beads	Chromotek	Cat# 141205001A
GFP-Trap Magnetic Agarose beads	Chromotek	Cat# 90312001MA
Tween 20	Acros Organics	Cat# 233362500
Proteinase K	Invitrogen	Cat# 1657252
Tris Ultrapure	Duchefa-Biochemie	Cat# 010894.04
Sodium acetate	Carlo Erba	Cat# 478137
Basic Fuchsine	BioPlus	Cat#2177006-1
Xylitol	Sigma-Aldrich	Cat# 87-99-0
Sodium deoxycholate	Sigma-Aldrich	Cat# 302-95-4
Urea	Acros Organics	Cat# 57-13-6
Propidium Iodide	Sigma-Aldrich	Cat# MKBV0241V

**Critical Commercial Assays**		

SensiFAST SYBR	Bioline	Cat# BIO-92005
NucleoSpin RNA Plus	Macherey-Nagel	Cat# 740984
qPCRBIO SyGreen Mix	PCR Biosystems	Cat# PB20.11-05
Gel/PCR DNA Fragments Extraction Kit	Geneaid	Cat# DF100
NucleoSpin Plasmid	Macherey-Nagel	Cat# 740588
Gateway BP Clonase II	Thermo-Fisher	Cat# 11789
Gateway LR Clonase II	Thermo-Fisher	Cat# 11791
Superscript VILO cDNA Synthesis Kit	Thermo-Fisher	Cat# 11754
Rneasy Micro Kit	QIAGEN	Cat# 74004
MinElute Reaction Cleanup Kit (50)	QIAGEN	Cat# 28204b

**Experimental Models: Organisms/Strains**		

*Arabidopsis: Col-0*	NASC	N/A
*Arabidopsis: phb-13,phv-11 ER+*	This paper	N/A
*Arabidopsis: cycd6-1*	NASC	SALK_021738
*Arabidopsis: CYCD6;1::GFP:GUS*	^5^	N/A
*Arabidopsis: UAS::PHBmu*	^9^	N/A
*Arabidopsis: Q0990*	NASC	N9217
*Arabidopsis: gai-t6*	^39^	N/A
*Arabidopsis: gai-1ER+,CYCD6;1::GFP:GUS*	This paper	N/A
*Arabidoopsis: CO2::His2B:YFP*	^13^	N/A
*Arabidopsis: gai-2*	NASC	SAIL_587_C02
*Arabidopsis: gai-3*	NASC	SALK_208684
*Arabidopis: GAI-GFP*	^40^	N/A
*Arabidopsis: GA2ox2::GUS*	^41^	N/A
*Arabidopsis: UAS:GA2ox2*	This paper	N/A
*Arabidopsis: UAS::MIM165/6*	This paper	N/A
*Arabidopsis: EN7::GAL4*	This paper	N/A
*Arabidopsis: phb-1d*	^12^	N/A
*Arabidopsis: ga2ox2-1*	NASC	SALK_051749
*Arabidopsis: RGA-GFP*	^32^	N/A
*Arabidopsis: phb-1d,RGA-GFP*	This paper	N/A
*Arabidopsis: phb-1d,GAI-GFP*	This paper	N/A
*Arabidopsis: PHB::GFP*	^12^	N/A
*Arabidopsis: PHB-GFP*	^12^	N/A
*Arabidopsis: scl3-1*	NASC	SALK_002516
*Arabidopsis: scl3-1,phb13,phv11*	This paper	N/A
*Arabidopsis: shr-2*	^42^	CS2972
*Arabidopsis: MIR165A::GFP*	^9^	N/A
*Arabidopsis: MIR166A::GFP*	^9^	N/A

**Oligonucleotides**		

See STAR Methods section and Tables S1–S3	N/A	N/A

**Recombinant DNA**		

*pB7m43GW*	^43^	N/A
*P4P1-UAS*	^43^	N/A
*P221-GA2OX2*	This paper	N/A
*P2P3-NOS*	^44^	N/A
*pDONR221-MIM165/6*	This paper	N/A
*pDONORP4P1-pEN7*	This paper	N/A
*pDONOR221-GAL4*	^43^	N/A

**Software and Algorithms**		

Excel	Microsoft	N/A
ImageJ	https://imagej.nih.gov/ij/	N/A
GraphPad	https://www.graphpad.com/scientific-software/prism/	N/A
PlantPAN2.0	http://plantpan2.itps.ncku.edu.tw/	N/A

**Other**		

Zen 2010	Zeiss	N/A
Fitotron SGC 120 Growth chamber	Weiss Technik, UK	N/A
Zeiss Axio Imager A2	Zeiss	N/A
7500 Fast Real-Time PCR system	Applied Biosystems	N/A
Branson Digital Sonifier 450	Fisher Scientific	N/A

### Resource Availability

#### Lead Contact

Further information and requests for resources and reagents should be directed to and will be fulfilled by the Lead Contact, Raffaele Dello Ioio (raffaele.delloioio@uniroma1.it).

#### Materials Availability

Unique materials used in this study will be freely available.

#### Data and Code Availability

This study did not generate any unique datasets or code.

### Experimental Model and Subject Details

*Arabidopsis thaliana* background lines Columbia-0 (Col-0) were used for experimentation, with mutants and transgenic lines in these backgrounds as detailed in the Key Resources Table.

### Method Details

#### Plant Material and Growth Conditions

The *Arabidopsis thaliana* ecotypes Columbia-0 (Col-0) was used as controls as the *gai-t6*,^45^ *gai-2*, *gai-3*, *phb-13, phv-11 ER+*,^12^ *phb-1d*,^12^ *cycd6;1-1*^5^, *ga2ox2-1* and *scl3-1* are in this background. *gai-1 ER+, CYCD6;1::GFP:GUS* was obtained by F3 populations of *gai-1 ER+, CYCD6;1::GFP:GUS* crosses. *ER+* was selected by phenotype. *scl3-1, gai-2*, *gai-3* and *ga2ox2-1* mutants were obtained from the NASC collection (SALK_002516, SAIL_587_C02, SALK_208684 and SALK_051749 respectively). Homozygous mutants from the Salk T-DNA were identified by PCR as described (http://signal.salk.edu/tdnaprimers.2.html). *gai-1* and *gai-t6* mutants were genotyped as described in Dill and Sung.^45^ *phb-13, phv-11 ER+* were obtained by crossing *phb-13, phv-11*^46^ with wild-type (Wt) Col-0 background. *ER+* was selected by phenotype. Enhancer trap line *Q0990* was obtained from the NASC. CO2::H2B:YFP, CYCD6;1::GFP:GUS, RGA-GFP and GAI-GFP transgenic plants have been described previously.^5^^,^^13^^,^^40^ *phb-1d,RGA-GFP, phb1-d,GAI-GFP*, *phb1-d,GA2ox2::GUS*, and *phb-13,phv-11,ER+,CYCD6;1::GFP:GUS* were obtained by crossing. UAS::PHBmu:GFP plants were obtained transforming the UAS::PHBmu:GFP plasmid^9^ in Wt Col-0 background via floral dip.^47^ For growth conditions, *Arabidopsis* seeds were surface sterilized, and seedlings were grown on _1/2_ Murashige and Skoog (MS) medium containing 0.8% agar at 22°C in long-day conditions (16-h-light/8-h-dark cycle) as previously described.^48^

#### MC analysis and confocal imaging

For root MC analysis, root meristems of 5 and 8 days post germination (dpg) plants were analyzed utilizing a differential Interference Contrast (DIC) with Nomarski technology microscopy (Zeiss Axio Imager A2). Plants were mounted in a chloral hydrate solution (8:3:1 mixture of chloral hydrate:water:glycerol). Confocal images were obtained using a confocal laser scanning microscope (Zeiss LSM 780). For confocal laser scanning analysis, the cell wall was stained with 10 μM propidium iodide (Sigma-Aldrich). For vascular analysis Basic fuchsin (BioPlus) was combined with Clearsee as described in Ursache et al.^49^ For each experiments, a minimum of 20 roots for three biological replicates were analyzed.

Data reported in histograms represent the average of the three biological replicates. MC formation frequency is calculated as percentage of plants presenting periclinal divisions in the endodermis. Statistics has been calculated utilizing GraphPad Prism Version (https://www.graphpad.com/scientific-software/prism/).

#### Generation and Characterization of Transgenic Plants

Standard molecular biology techniques and the Gateway system (Invitrogen) were used for the cloning procedures. For the UAS::GA2ox2 transgenic plant, the genomic sequence of GA2ox2 (2450 bp) was amplified from genomic DNA of *Arabidopsis* Columbia-0 ecotype using specific primers (GA2ox2 FW 5′- GGGGACAAGTTTGTACAAAAAAGCAGGCTGGATGGTGGTTTTGCCACAGC −3′, GA2ox2 REV 5′- GGGGACCACTTTGTACAAGAAAGCTGGGTGTCATACAAGGGTTTTATGATTGAG −3′) and cloned in a pDONOR221 (pDONOR221-gGA2ox2). A LR reaction was then conducted by using the pDONORP4P1-UAS, pDONOR221-gGA2ox2 and a pDONORP2P3-NOS vector. The LR products were sub-cloned in the Gateway pBm43GW destination vector. Plasmids were transformed into Q0990 plants by floral dipping.^44^

For *EN7>>MIM165/6* transgenic plant, *UAS::MIM165/6* transcriptional fusion was obtained as follow: the sequence of MIM165/166 was amplified from vector generated by Todesco et al.^48^ using specific primers (MIM165FW 5′-GGGGACAAGTTTGTACAAAAAAGCAGGCTGGGG CCGCAAAACACCACAAAAACA-3′, MIM165REV 5′-GGGGACCACTTTGTACAAGAAAGC TGGGTGAACTAGTGGATCCCCCATCACCAC-3′) and cloned in *pDONR221* Gateway vector by BP recombination (Invitrogen). Subsequently *pDONRP4P1-UAS*, *pDONR221-MIM165/6* and *pDONR P2P3-NOS* were recombined into a *pB7m34GW* destination vector via LR reaction (Invitrogen). To generate *EN7::GAL4* construct, *pDONORP4P1-pEN7* and *pDONOR221-GAL4* were recombined with *pDONOR P2P3-NOS* into a *pB7m34GW* destination vector via LR reaction (Invitrogen). Plasmids were transformed into Col-0 plants by floral dipping.^47^ Then, *EN7::GAL4* plants were crossed with *UAS::MIM165/6* plants.

#### Drug treatments

3 dpg seedlings were transferred with tweezers onto solid _1/2_ MS medium plates containing PAC (PACLOBUTRAZOL) (Duchefa) at a final concentration of 50 μM or GA_4+7_ (Gibberellin _4+7_, Duchefa) at a final concentration of 100 μM for 24 and 48 hours depending on the experiment (see legend).

#### GUS histochemical assay

*β-Glucuronidase* activity of transgenic lines carrying the GUS enzyme was assayed essentially as described in Moubayidin et al.^12^ using the β-glucoronidase substrate X-GlcA, (5-Bromo-4-chloro-3-indolyl-β-D-glucuronic acid, Duchefa) dissolved in DMSO. X-GlcA solution: 100 mM Na_2_HPO_4_, 100 mM NaH_2_PO_4_, 0.5 mM K3 K_3_ Fe(CN)_6_, 0.5 mM K_4_Fe(CN)_6_, 0.1% Triton X-100 and 1 mg/ml X-GlcA. Seedlings were incubated at 37°C in the dark for an appropriate time allowing tissue staining depending on the GUS line assayed. Imaging was done using the Axio Imager A2 (Zeiss) microscopy. For each line and time-point, at least 20 roots were analyzed and the percentages of phenotypes were evaluated.

#### RNA isolation, reverse-transcription and qRT-PCR

Total RNA was extracted from 5 dpg or 8 dpg old roots using the NucleoSpin RNA Plus (Macherey-Nagel). The cDNA was retro-transcribed using the SuperScript III First-Strand VILO cDNA Synthesis Kit (ThermoFisher Scientific). Quantitative RT-PCR (qRT-PCR) analysis were performed using the gene-specific primers listed in Table S2. All primers are given in the 5′-to-3′ direction. All the primers were tested for their qPCR efficiency of 2-fold amplifications per cycle by qRT-PCR with the Standard curve method. PCR amplifications were carried out using the SensiFast SYBR Lo-Rox (Bioline) mix. Amplification was monitored in real time with a 7500 Real Time PCR System (Applied Biosystems). Amplification of *ORNITHINE TRANSCARBAMYLASE (OTC)* and *GLYCERALDEIDE-PHOSPHATE-DEHIDROGENASE* (*GAPDH)* served as housekeeper controls. Data are expressed in 2^-ΔΔct^ value. Three technical replicates of qRT-PCR were performed on two independent RNA batches. Results were comparable in all the experiments and with both housekeepers. Student’s t test was performed to assess the significance of the differences between each sample and the control sample. In figures are reported data normalized to OTC. In Figures 1P and 4A the normalization base is 5 dpg.

#### MIR165a/166a Fluorescence Quantification

The fluorescence value of *MIR165A::GFP* and *MIR166A::GFP* (Figure S1) was obtained as reported in Di Mambro and Sabatini.^48^ The plugin *MeasureRGB* of the software *ImageJ* (https://imagej.nih.gov/ij/) quantify the Σ of pixels of the channel (raw intensity density—*RawIntDen*). *RawIntDen* values of GFP channel of confocal microscope images were obtained taking into consideration the same area for 5 and 8 dpg of *MIR165A::GFP* and *MIR166A::GFP*, respectively, starting from the QC, keeping the same acquisition setting for both 5 and 8 dpg. Student’s t test was used to determine the statistical significance (https://graphpad.com:443/quickcalcs/ttest2.cfm) as reported in the relative figure legend.

#### ChIP-qPCR analysis

ChIP was conducted following modified protocols from Lawrence et al.^50^ and Kaufmann et al.^51^ on 2-3 biological replicates of *PHB::GFP* (Col-0), as control, and *PHB-GFP* (Col-0) roots at 8 dpg.

0.8-1.5g of roots were harvested in 50ml collection tubes and cooled on ice. Tubes were covered with Miracloth (Merk Millipore) and tissues were rinsed twice with 40ml of ddH_2_O. Plant material was fixated with 37ml of ddH_2_O and 1ml of 37% (w/v) formaldehyde on ice. Then, vacuum was applied for ten minutes. Vacuum was slowly released and material was mixed inverting the tubes gently. After five minutes vacuum was re-applied for another ten minutes. This step was repeat three times. To quench crosslinking, 2.5ml (1.25M stock) of glycine (Biorad) was added and vacuum was applied for five minutes. The vacuum was released slowly and plant material was rinsed twice with ddH_2_O. The plant material was dried between two tissue layers and quick-frozen in liquid nitrogen. Then plant material was ground to a fine powder and placed into a pre-cooled 50ml tube.

30 mL of ice-cold Extraction Buffer 1 (0.4M sucrose, 10 mM TRIS-HCl pH 8.0, 10 Mm MgCl_2_, 5 mM DDT, protease inhibitor cocktail) was added to the material and immediately vortexed until a homogeneous mixture was obtained. Tubes were kept on ice on 30 minutes. The solution was filtrated twice through Miracloth (Merk Millipore) and centrifuged for 15 minutes (4000 rpm, 4°C). The supernatant was gently discarded and the pellet was re-suspended in 1ml of Extraction Buffer 2 (0.25 sucrose, 10 mM TRIS-HCl pH 8.0, 10 Mm MgCl_2_, 0.15% Triton X-100, 5 mM DTT, protease inhibitor cocktail). Samples were centrifuged for 12 minutes (10000 rpm, 4°C). The pellet was re-suspended in 300 μl of Extraction buffer 2. 300 μl of Extraction Buffer 3 (1.7 M sucrose, 10 mM TRIS-HCl pH 8.0, 2 mM MgCl_2_, 0.15% Triton X-100, 5 mM DTT, protease inhibitor cocktail) was added and pellet was carefully layered upon it. Tubes were centrifuged for one hour (13000 rpm, 4°C) and the supernatant was carefully removed. This step permits the separation of two phases were nuclei are suspended in the pellet. The pellet was re-suspended in 500 μl of Nuclei Lysis Buffer (50 mM TRIS-HCl pH 8.0, 10 mM EDTA, 1% (v/v) SDS, protease inhibitor cocktail). Chromatin was sonicated with a probe sonicator (Brenson) for 3 cycles of 5 s of a 25% of power and 5 s of cooling between the pulses. Samples were cooled for 4 minutes and re-sonicated for 6 cycles for 5 s of 28% of power and 5 s of cooling between the pulses. Tubes were placed on ice the whole time. The sonication allows to obtain fragments of approximately 600 bp. The tubes were centrifuged for ten minutes (13000 rpm, 4°C) and the supernatant was transferred into new 2 mL safe lock tubes. This step was repeated and the supernatant (0.4 ml) was transferred to 15 mL falcon tubes containing 3.6 mL of ChIP Dilution Buffer (1% (v/v) Triton X-100, 1.2 mM EDTA, 16.7 mM TRIS-HCl (pH 8.0), 167 mM NaCl) (1:10 dilution). 120 μl of sample was set aside as input DNA control.

To preclear chromatin, first of all, 30 μl of blocked agarose beads (Chromoteck, Planegg-Martinsried, Germany) were suspended in 10 mL of ChIP Dilution Buffer ad mixed. The tubes were centrifuged for two minutes (2500 rpm, 4°C) and the supernatant was discarded. This wash step was repeated twice. At this point, chromatin was mixed with the blocked agarose beads and incubated at 4°C for one hour. In the meantime, 40 μl of GFP-trap agarose beads and 40 μl of blocked agarose beads (Chromotek, Planegg- Martinsried, Germany) were suspended, separately, in 1 mL of ChIP Dilution Buffer into new 2 mL safe lock tubes. The beads were centrifuged for three minutes (2500 rpm, 4°C) and supernatant was carefully discarded. This wash step was repeated twice. Afterward, chromatin was centrifuged for three minutes (2500 rpm, 4°C) and the supernatant was carefully transferred to a new pre-cooled 15 mL falcon tube, taking care to transfer any beads. This step was repeated and the supernatant was transferred to a new pre-cooled 15 mL tubes. At this point the resulting 4 mL of samples were divided into 2 mL aliquots: one aliquot was added to the GFP-trap agarose beads and the other to the blocked agarose beads, as a negative control. The samples were incubated for the IP at 4°C overnight on a rotating wheel.

Samples were centrifuged for three minutes (2500 rpm, 4°C) and the supernatant was removed. Then, 1 mL of Low salt Buffer (150 Mm NaCl, 0.1% SDS, 1% (v/v) Triton X-100, 2 mM EDTA, 20 mM TRIS-HCl pH 8.0) was added in the tubes and beads were incubated at 4°C on a rotating wheel for seven minutes and, then, centrifuged for three minutes at 2500 g at 4°C. This wash step was repeated utilizing, in order, the following buffer: High Salt Buffer (500 mM NaCl, 0.1% SDS, 1% (v/v) Triton X-100, 2 mM EDTA, 20 mM TRIS-HCl pH 8.0) and TE Buffer (10 mM TRIS-HCl pH 8, 1 mM EDTA). The TE Buffer wash was performed three times.

To elute the protein-DNA complex from the beads 100 μl of cold Elution Buffer (0.1 M glycine, 0.5 M NaCl, 0.05% Tween-20, pH was adjusted to 2.8) was added to the samples, which were immediately vortexed and incubated for one minutes at 37°C while shaking vigorously. Tubes were centrifuged for one minutes (13000 rpm, room temperature) and the supernatant (eluate) was transferred to a new safe lock 2 mL tubes, where 50 μl of TRIS-HCL (1 M stock, pH 9.0) was added to neutralize it. The elution step was repeated incubating tubes, with remaining protein-DNA complex, for two minutes at 37°C while shaking vigorously. Tubes were centrifuged for one minute (13000 rpm, room temperature) and supernatant were transferred to the eluate of the first elution. 50 μl of TRIS-HCl (1 M stock, pH 9.0) was added to neutralize. The elution step was repeated incubating tubes at 37°C while shaking vigorously and spinning for one minutes (13000 rpm, room temperature). The supernatant was combined with previously eluates. 50 μl of TRIS-HCl (1 M stock, pH 9.0) was added to neutralize, obtaining a final volume of 450 μl. The samples were centrifuged for two minutes (13000 rpm, room temperature) and the supernatant was transferred to new 2 mL safe lock tubes, taking care to disintegrate any pellet that may have been formed.

12.5 μl of proteinase K (18 mg/ml stock; final concentration should be 0.5 mg/ml) was added to the eluates, which were incubated at 37°C overnight to reverse crosslinking. A second aliquot of proteinase K (same amount) was added to the samples and the tubes were incubated at 65°C for six hours.

DNA was purified with the MinElute PCR purification kit (Quiagen, Venlo, NL). The total volume (472.5 μl) of the eluted DNA was split in two aliquots and each of them was mixed with 1181,25 μl of Binding Buffer PB, provided by the kit, and 30 μl of sodium acetate (3 M stock, pH 5.0). At this point, kit instructions were followed. The elution step was performed incubating for five minutes DNA with 25 μl of ddH_2_O. Then, DNA was centrifuged for 30 s (13000 rpm, room temperature). This step was repeated. The total volume (50 μl) was diluted with 50 μl of ddH_2_O to perform qRT-PCR analysis.

qRT-PCR was performed using the 7500 Real Time PCR System (Applied Biosystems). Primers (Table S3), spannig three region of *GA2ox2* promoter, were tested for their qPCR efficiency of 2-fold amplifications per cycle by Standard curve method. PCR amplifications were carried out using the SensiFast SYBR Lo-Rox (Bioline) mix. Analysis was performed in triplicates from 2-3 independent chromatin immunoprecipitations. The fold enrichment of fragments was obtained as ratio of anti-GFP IP to control beads IP of each independent replicate. *UBQ10* (UBQ10-F 5′-GGCCTTGTATAATCCCTGATGAATAAG-3′, UBQ10-R 5′- AAAGAGATAACAGGAACGGAAACATAGT −3′) was used for normalization. Primers are given in the 5′-to-3′ direction.

#### Seeds sterilization protocol for ChIP

In 50 mL falcon tubes seeds were mixed for five minutes with a solution composed by bleach 50% and Tween 10%. Seeds were centrifuged for one minute (410 g, room temperature) and supernatant was discarded. H_2_O was added to seeds. They were mixed for five minutes and centrifuged for one minute (410 g, room temperature). This wash step was repeated for other four times (five total washes).^48^ Seeds were dried under sterile flux and they were stratified with agarose 0.1% in darkness at 4°C for three days. Then, they were grown on solid _1/2_ MS medium at 22°C in long-day conditions (16-h-light/8-h-dark cycle).

### Quantification and Statistical Analysis

Statistical analysis was performed using GraphPad (https://www.graphpad.com/scientific-software/prism/). In all plots, error bars represent standard deviations (SD). The significance of the data was evaluated using the Student’s t test (^∗^p < 0,05, p ^∗∗^ < 0,01, p^∗∗∗^ < 0,005, NS Not Significant). For the statistical analysis of the MC frequency percentage was performed a one-way ANOVA analysis with post hoc Dunnet testing. Significantly different groups of samples are indicated using lower case letters.

GFP fluorescence signal intensity was measured and quantified with the *ImageJ* (https://imagej.nih.gov/ij/) software.

All experiments have been performed in at least three replications, using enough number of samples to ensure statistical significance.

## Supplemental Information

Document S1. Figures S1–S4 and Tables S1–S3

Document S2. Article plus Supplemental Information
