# Supplementary material for: A PHABULOSA-Controlled Genetic Pathway Regulates Ground Tissue Patterning in the Arabidopsis Root
Source: Curr Biol. 2021 Jan 25;31(2):420–426.e6. doi: 10.1016/j.cub.2020.10.038 (PMC7846283; doi:10.1016/j.cub.2020.10.038)
Supplement: Document S1. Figures S1–S4 and Tables S1–S3 [file mmc1.pdf]

Current Biology, Volume *31*

Supplemental Information

A PHABULOSA-Controlled Genetic Pathway Regulates Ground Tissue Patterning in the *Arabidopsis* Root

Gaia Bertolotti, Simon Josef Unterholzner, Daria Scintu, Elena Salvi, Noemi Svolacchia, Riccardo Di Mambro, Veronica Ruta, Francisco Linhares Scaglia, Paola Vittorioso, Sabrina Sabatini, Paolo Costantino, and Raffaele Dello Ioio

SUPPLEMENTAL DATA:

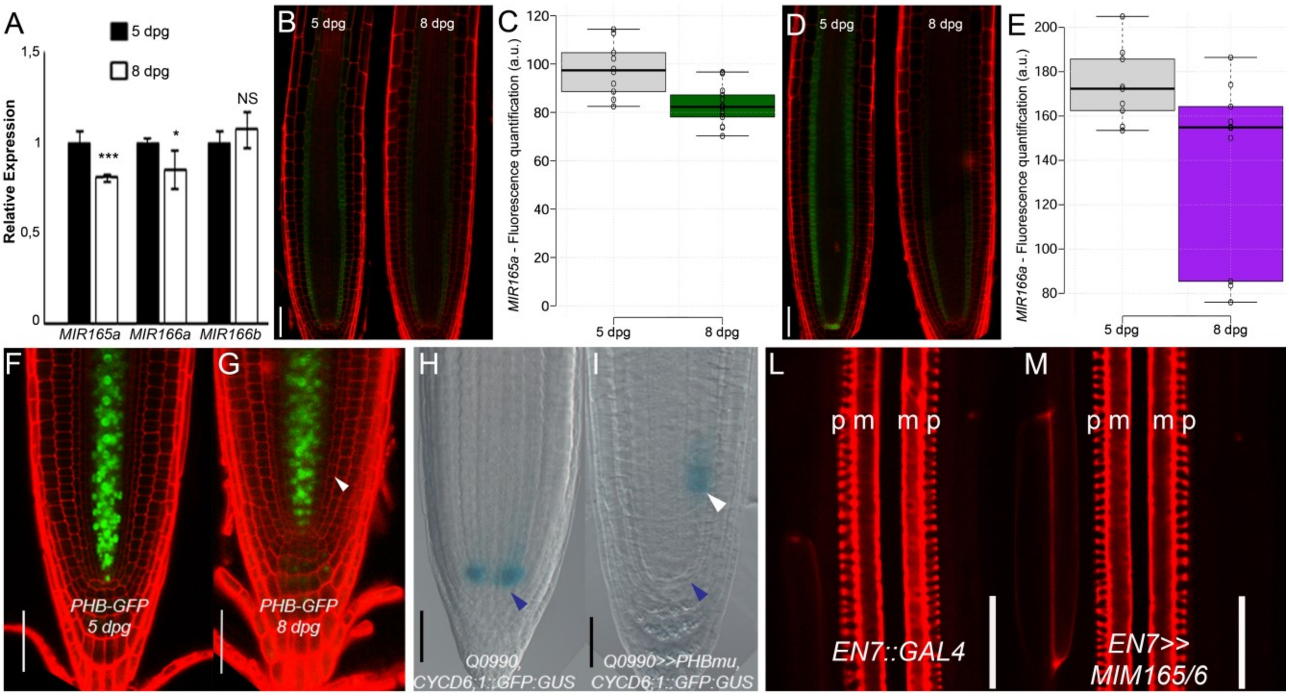

SD1: PHB directs *CYCD6;1* expression switch cell non autonomously. Related to Figure 1.
A) Relative expression of *pre-miR165a, pre-miR166a* and *pre-miR166b* in 5 and 8 dpg Wt plants. N=3, Student t test (*p<0,05, p **<0,01, p***<0,005, NS Not Significant). Error Bars: SD. B) Confocal images of *MIR165A::GFP* roots at 5 dpg and 8 dpg. Scale Bars: 50 µm. C) Fluorescence quantification of *MIR165A::GFP* line at 5 dpg (grey) and at 8 dpg (green) where center lines show the medians. Box limits indicate the 25th and 75th percentiles as determined by R software. Whiskers extend 1.5 times the interquartile range from the 25th and 75th percentiles; data points are plotted as open circles. p-value < 0.005, Student's t-test, n = 10, 14 sample points. D) Confocal images of *MIR165A::GFP* roots at 5 dpg and 8 dpg. Scale Bars: 50 µm. E) Fluorescence quantification of *MIR166A::GFP* line at 5 dpg (grey) and at 8 dpg (purple) where center lines show the medians. Box limits indicate the 25th and 75th percentiles as determined by R software. Whiskers extend 1.5 times the interquartile range from the 25th and 75th percentiles; data points are plotted as open circles. p-value < 0.05, Student's t-test, n = 9, 10 sample points. F,G) Confocal image of a root meristem of a *PHB-GFP* plant at 5 (F) and 8 dpg (G). H,I) Optical microscope images of a 5 dpg root meristem of *Q0990, CYCD6;1::GFP:GUS* (H) and *Q0990>>PHBmu:GFP, CYCD6;1::GFP:GUS* (I). Scale Bars: 50 µm, white arrowheads indicate MC formative asymmetric division, blue arrowheads CEI. L,M) Confocal images of 5 dpg basic fuchsin-clearsee stained *EN7::GAL4* (L) and *EN7>>MIM165/6* (M) roots. p: protoxylem, m:metaxylem. Scale Bars: 20 µm.

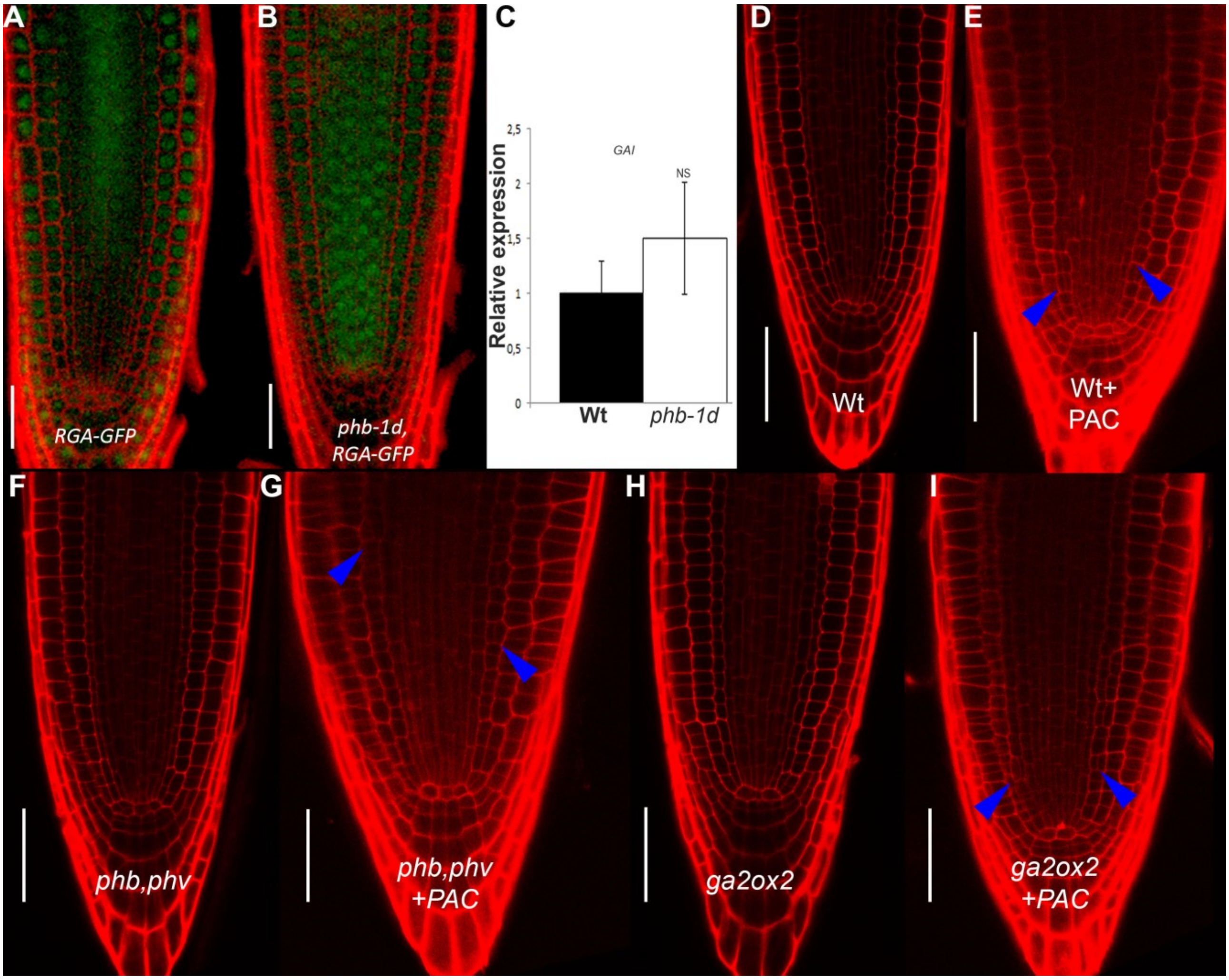

SD2: PHB regulates MC formation via GAs metabolism regulation. Related to Figure 2.

A-B) Confocal images of 5 dpg root meristems of *RGA-GFP* (A) and *phb-1d, RGA-GFP* (B). C) qRT-PCR of *GAI* in Wt and *phb-1d* mutants. N=3, Student t test (NS Not Significant). Error Bars: SD. D-I) Confocal images of 5 dpg root meristems of Wt (D), Wt+ PAC (50 μM 48h) (E), *phb, phv* (F), *phb, phv*+ PAC (50 μM 48h) (G), *ga2ox2* (H),*ga2ox2*+ PAC (50 μM 48h)(I). Scale Bars: 50 μm, blue arrowheads indicate the additional asymmetric division induced by PAC.

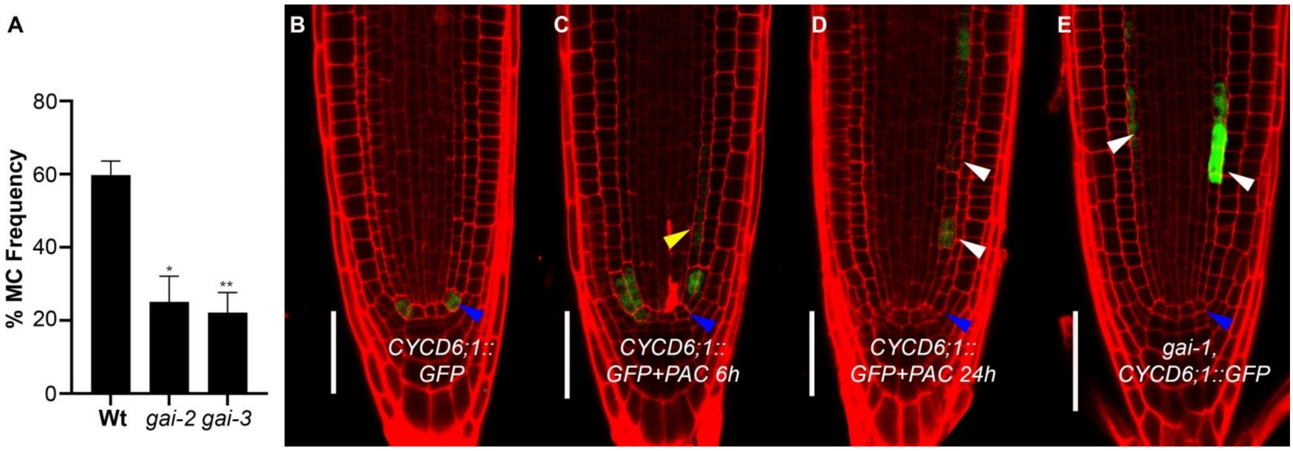

SD3: GAI directs *CYCD6;1* expression switch. Related to Figure 3

A) Histogram depicting the percentage of plants showing MC in Wt and *gai-2* and *gai-3* at 8 dpg N=3, Student t test (*p<0,05, p **<0,01). Error Bars: SD. ANOVA p<0,01. B-E) Confocal images 5 dpg the root meristem of *CYCD6;1::GFP:GUS* untreated Wt (B), treated (C-D) with PAC (50 μM) for 6 (C) and 24 (D) hours and *gai-1* mutant (E). Scale Bars: 50 μm, white arrowheads indicate MC formative asymmetric division, blue arrowheads CEI, yellow arrowhead shows the cells accumulating GFP signal prior to cell division.

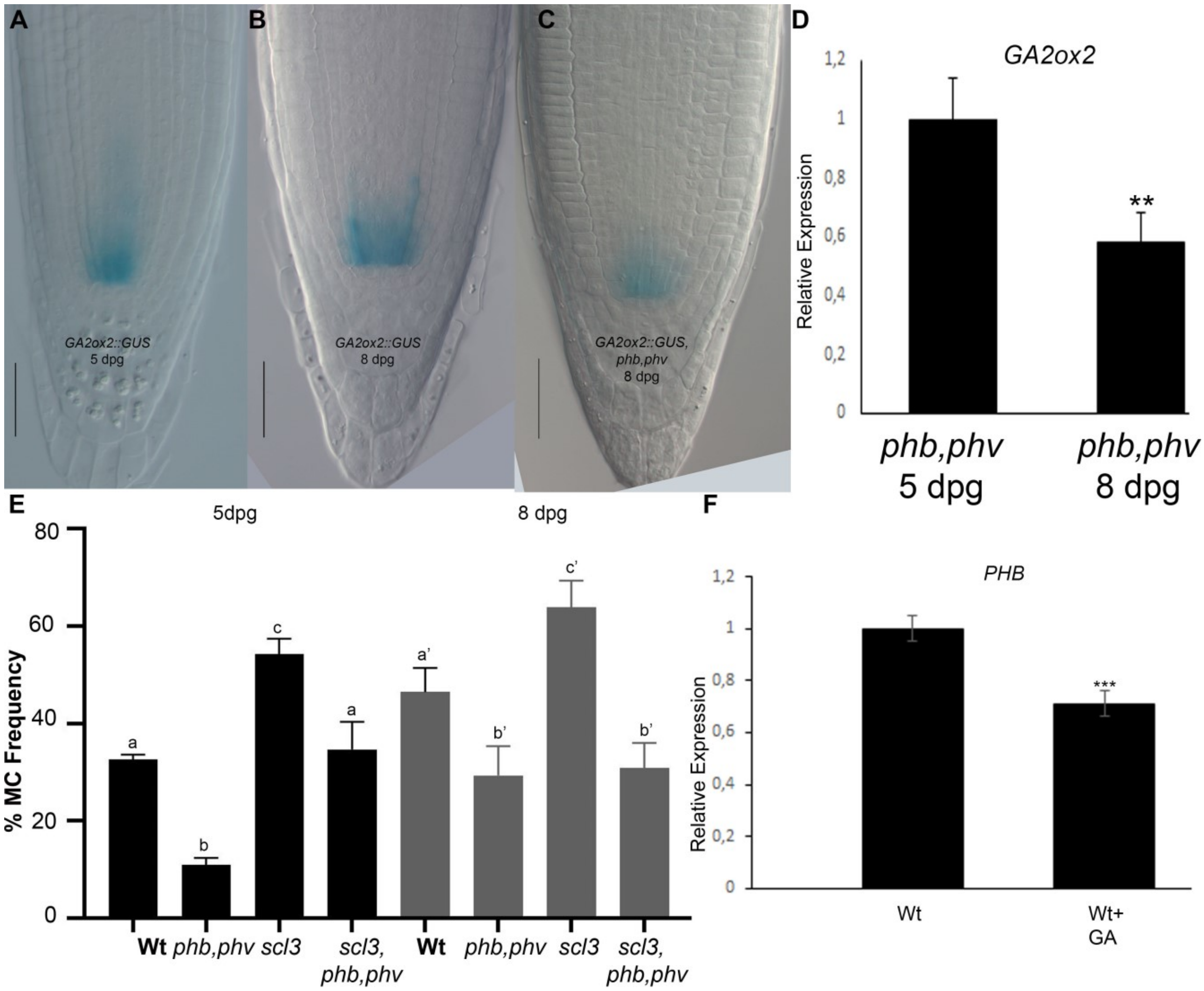

SD4: PHB and PHV dependent GAs catabolism in the vasculature control MC formation via SCL3. Related to Figure 4.

A-C) Root meristems of *GA2ox2::GUS* plants at 5 (A) and 8 (B) dpg and 8 dpg *GA2ox2::GUS,phb,phv* plants (C). Scale Bars: 50 μm. D) qRT-PCR of *GA2ox2* in *phb,phv* mutants at 5 and 8 dpg. N=3, Student t test (p **<0,01). Error Bars: SD. E) Histogram depicting the percentage of plants showing MC in Wt and *phb,phv* and *scl3* and *scl3,phb,phv* at 5 and 8 dpg. N=3, Student t. Error Bars: SD. P<0,05 ANOVA. Letters indicate statistical significance. F) qRT-PCR of *PHB* in Wt plants untreated and treated with GA3 for 6 hours at 8 dpg. N=3, Student t test (p ***<0,05). Error Bars: SD.

Genotyping primers

Background	Forward	Reverse	Insertion
phb-13	ACCGTACCCTGGATTTAG CC	TTATCTAGATCCGGTGGATCC AAG	TCAAACGAACGACCAATTC ACG
phv-11	CCCAATGGTCCACTTTCTT CA	GCCAGCAAATTTAGCAGAGGA	GCGTGGACCGCTTGCTGC AACT
gai-t6	CTAGATCCGACATTGAAG GA	AGCATCAAGATCAGCTAAAG	TCGGTACGGGATTTTCGCA T
gai-1	CTAGATCCGACATTGAAG GA	AGCATCAAGATCAGCTAAAG	AGCATCAAGATCAGCTAAA G
cycd6-1	AATTCGACGACCCATCTCT G	CTGCAATCACCGATGGTTTA	ATATTGACCATCATACTCA TTGC
gai-2	TGTACCACTAGTTGCATG ACAATC	AGCTTCGGCGAAGTAAGTAGC	GCCTTTTCAGAAATGGATA AATAGCCTTGCTTCC
gai-3	TCGATAAGGTTCTTGGTG TGG	CAAAGGGTCACGAGTGAAGTC	GCGTGGACCGCTTGCTGC AACT
ga2ox2-1	GAAAACCCGAATCGTAAA AGC	GAGACGAGAAGAAATCGCAT G	GCGTGGACCGCTTGCTGC AACT
scl3-1	AGCGCAGTTCTTTCTCATG AG	TTCCTCTGTTCTTTAACCCCC	GCGTGGACCGCTTGCTGC AACT

Table S1: Genotyping primers used in this study. Related to STAR Methods

qRT-PCR primers

Gene	Forward	Reverse
GADPH	TTGGTGACAACAGGTCAAGCA	AAACTTGTCGCTCAATGCA
OTC	TGAAGGGACAAAGGTTGTGTATGTT	CGCAGACAAAGTGGAAT GGA
PHB	GCTAACAACCCAGCAGGACTCCT	TAAGCTCGATCGTCCCACCGTT
GA2ox2	TCCGACCCGAACTCATGACT	CGGCCCGGTTTTTAAGAGAC
GAI	CTAGATCCGACATTGAAGGA	AGCATCAAGATCAGCTAAAG
PHV	GCTAATCTTCTCTCGATTGCGGAGGA	GCTCGATAGTACCACCATTTCCAGTG
MIR165A	GATCGATTATCATGAGGGTTAAGC	CTATAATATCCTCGATCCAGACAAC
MIR166A	GGGGCTTTCTCTTTTGAGG	CGAAAGAGATCCAACATGAATAG
MIR166B	GATTTTTCTTTTGAGGGGACTGTTG	CTGAATGTATTCAAATGAGATTGTATTAG

Table S2: qRT-PCR primers used in this study. Related to STAR Methods

ChIP-qPCR primers

Region	Forward	Reverse
A	CTGATCCATAGGCATCATGTA	TGCTTTCTGGTTGTAGGTTCTC
B	AGATCCAATCATTTTCCTCCAT	GTTTGGATAATTGGAAGAATTTTA
C	GACGGGCATTGGTGATTTATT	ATAGAAACATCCTTATCCTCAC

Table S3. ChIP-qPCR primers used in this study. Related to STAR Methods.
